# Identification of key candidate genes and molecular pathways in white fat browning: an anti-obesity drug discovery based on computational biology

**DOI:** 10.1186/s40246-019-0239-x

**Published:** 2019-11-07

**Authors:** Yuyan Pan, Jiaqi Liu, Fazhi Qi

**Affiliations:** Department of Plastic and Reconstructive Surgery, Zhongshan Hospital, Fudan University, 180 Fenglin Rd, Shanghai, 200032 People’s Republic of China

**Keywords:** Brown fat, Browning of white fat, PPAR-γ, β3-AR, Text mining, In silico, Drug therapy, Obesity

## Abstract

**Background:**

Obesity—with its increased risk of obesity-associated metabolic diseases—has become one of the greatest public health epidemics of the twenty-first century in affluent countries. To date, there are no ideal drugs for treating obesity. Studies have shown that activation of brown adipose tissue (BAT) can promote energy consumption and inhibit obesity, which makes browning of white adipose tissue (WAT) a potential therapeutic target for obesity. Our objective was to identify genes and molecular pathways associated with WAT and the activation of BAT to WAT browning, by using publicly available data and computational tools; this knowledge might help in targeting relevant signaling pathways for treating obesity and other related metabolic diseases.

**Results:**

In this study, we used text mining to find out genes related to brown fat and white fat browning. Combined with biological process and pathway analysis in GeneCodis and protein-protein interaction analysis by using STRING and Cytoscape, a list of high priority target genes was developed. The Human Protein Atlas was used to analyze protein expression. Candidate drugs were derived on the basis of the drug-gene interaction analysis of the final genes. Our study identified 18 genes representing 6 different pathways, targetable by a total of 33 drugs as possible drug treatments. The final list included 18 peroxisome proliferator-activated receptor gamma (PPAR-γ) agonists, 4 beta 3 adrenoceptor (β3-AR) agonists, 1 insulin sensitizer, 3 insulins, 6 lipase clearing factor stimulants and other drugs.

**Conclusions:**

Drug discovery using in silico text mining, pathway, and protein-protein interaction analysis tools may be a method of exploring drugs targeting the activation of brown fat or white fat browning, which provides a basis for the development of novel targeted therapies as potential treatments for obesity and related metabolic diseases.

## Introduction

Obesity is a global public health issue [1]. Excessive consumption of energy and the lack of exercise have increased the prevalence of obesity throughout the world. Statistically, based on the US population, 35% of adult men and 37% of adult women are obese [2]. This dramatically increases the risk of obesity-associated metabolic diseases, such as hyperglycemia, insulin resistance, type 2 diabetes, hyperlipidemia, hypertension, cardiovascular and cerebrovascular diseases, and other life-threatening diseases [3, 4]. The current treatment approaches for obesity are to reduce sugar intake and increase energy expenditure, including diet, exercise, medicine, and surgery. However, the therapeutic effect is not satisfactory. As a consequence, it is empirical to discover an effective and safer way to treat obesity. This may also help to prevent other metabolic diseases.

For a condition as dangerous as obesity, obesity is a dangerous condition and surprisingly, we know a little about its causes and its prevention. In recent years, the gradual in-depth understanding of the adipose tissue differentiation mechanism has provided new therapeutic targets for the development of obesity treatment. Adipose tissues, which are mainly composed of white adipose tissue (WAT), beige fat, and brown adipose tissue (BAT), are important in energy storage and regulation. WAT is the largest energy reservoir in the body, mainly storing energy in the form of triacylglycerol (TG) and release free fatty acids (FAs) in the fasting state for the maintenance of the metabolism and the other activities of the body. Compared with white adipocytes, fat in brown adipocytes usually store TG in the form of small fat droplets, while in white adipocytes, it is stored as large fat droplets. In addition, brown adipocytes are rich in mitochondria, the content of which far exceeds that of white adipocytes. In addition, there is a unique uncoupling protein 1 (UCP1) present on the mitochondrial inner membrane. A large number of protons in the intermembrane space are transferred into the mitochondrial matrix, which uncouples oxidative phosphorylation, thereby reducing mitochondrial membrane potential, increasing mitochondrial electron transport rate, and ultimately leading to heat production and consumption of large amounts of chemical energy [5].

Previously, human BAT was thought to exist only in newborns and regress with increasing age and become less related to metabolism in adults. However, several independent research teams confirmed their presence and function in the human body through the technology of positron emission tomography-computed tomography (PET-CT) [6]. This led to the discovery of beige fat which can appear in response to cold exposure, mainly distributed in subcutaneous WAT, specifically in inguinal WAT [7]. Under normal conditions of the human body, beige adipocytes are similar to white adipocytes, containing a large single-chamber lipid droplet, and the intracellular level of UCP1is low [8]. Beige adipocytes appear as multi-cavity lipid droplets and increased UCP1 levels, producing heat and thus transforming into a brown fat phenotype in response to cold exposure and certain other stimuli, such as β adrenoceptor receptor agonist stimulation. This process is called “browning of white fat” [9, 10]. Studies have shown that white, beige, and brown adipocytes are characterized by distinct lipidome, and the activation of beige fat and BAT can promote energy consumption and inhibit obesity, as well as modulating glucose and lipid metabolism disorders [11, 12]. Therefore, the activation of BAT or white fat browning is considered as an attractive drug target to promote weight loss and treat related metabolic diseases.

While the existing drug efficacy is limited and the traditional approach to new drug discovery is time-consuming and costly, drug repositioning, which refers to the identification of new indications from existing drugs and the newly identified drugs, may serve as a new way to treat diseases more effectively and less expensive [13]. This study aimed to investigate therapeutic targets and new drug therapies for the activation of BAT and browning of WAT, based on the work of related genes excavation by using computational methods to mine public databases and utilize bioinformatics tools to systematically identify interaction networks between drugs and gene targets, which provides experimental basis for an in-depth understanding of the mechanism of fat metabolism and explores new methods of obesity treatment. In this study, we used text mining to explore genes related to white fat browning. Combined with biological process and pathway analysis, protein-protein interaction analysis, a list of high priority target genes was picked out [14, 15]. By using a molecular tool, the expression profiles of candidate proteins in different tissues through immunohistochemical analysis were shown to verify whether a protein is truly expressed in the particular tissue or not. Based on the drug-gene interaction analysis of the final genes, candidate drugs were selected.

## Results

### Results of text mining, biological process, and pathway analysis

**Fig. 1:**
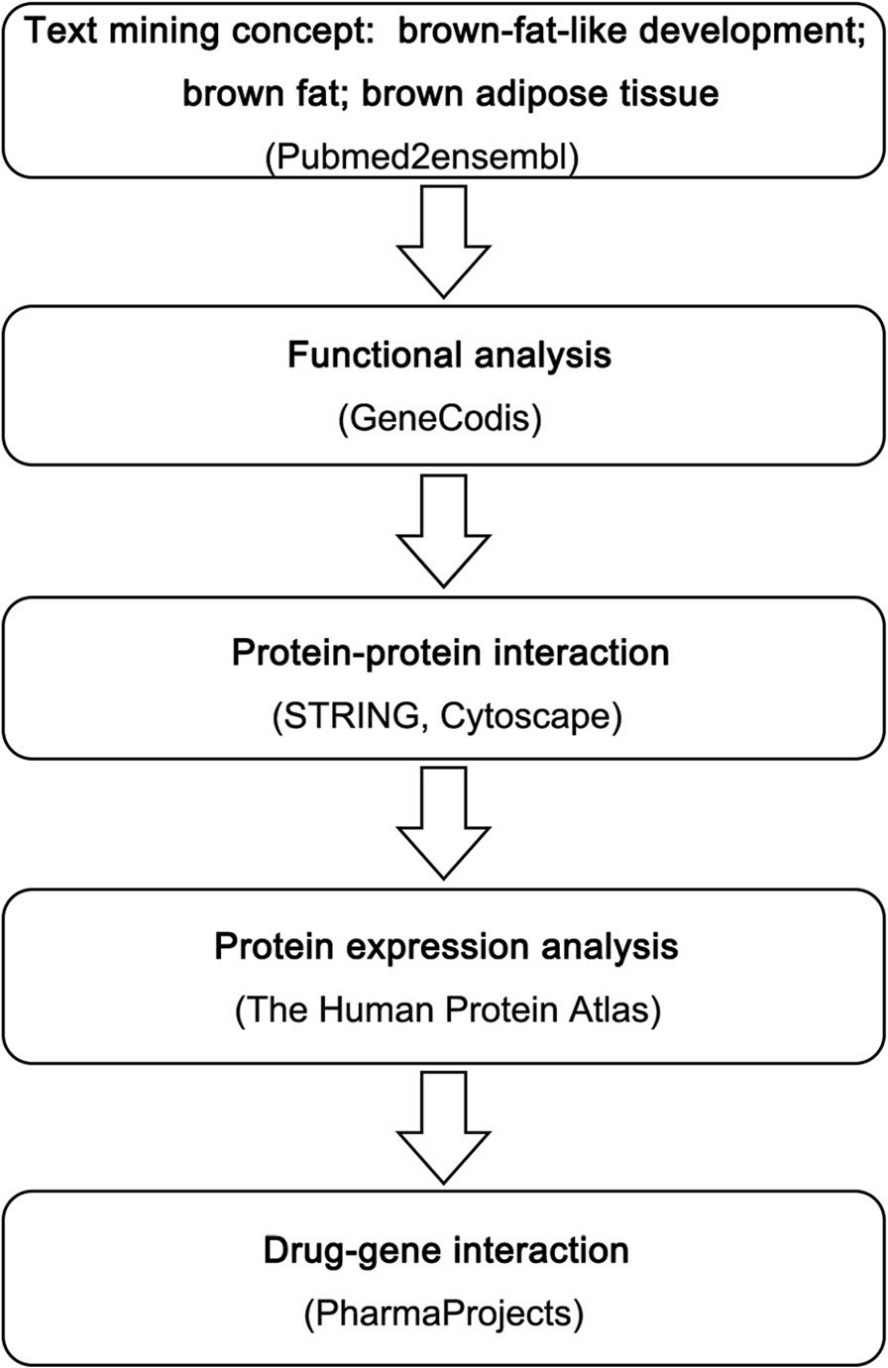
Overall data mining process. Text mining was used to identify genes associated with brown fat and white fat browning by using pubmed2ensemble. Combining with biological process and pathway analysis in GeneCodis and protein-protein interaction analysis by using STRING and Cytoscape, a list of high priority target genes was picked out. The Human Protein Atlas was used to analyze protein expression. The final drug list was obtained based on the gene-drug interaction analysis by using Pharmaprojects

**Fig. 2:**
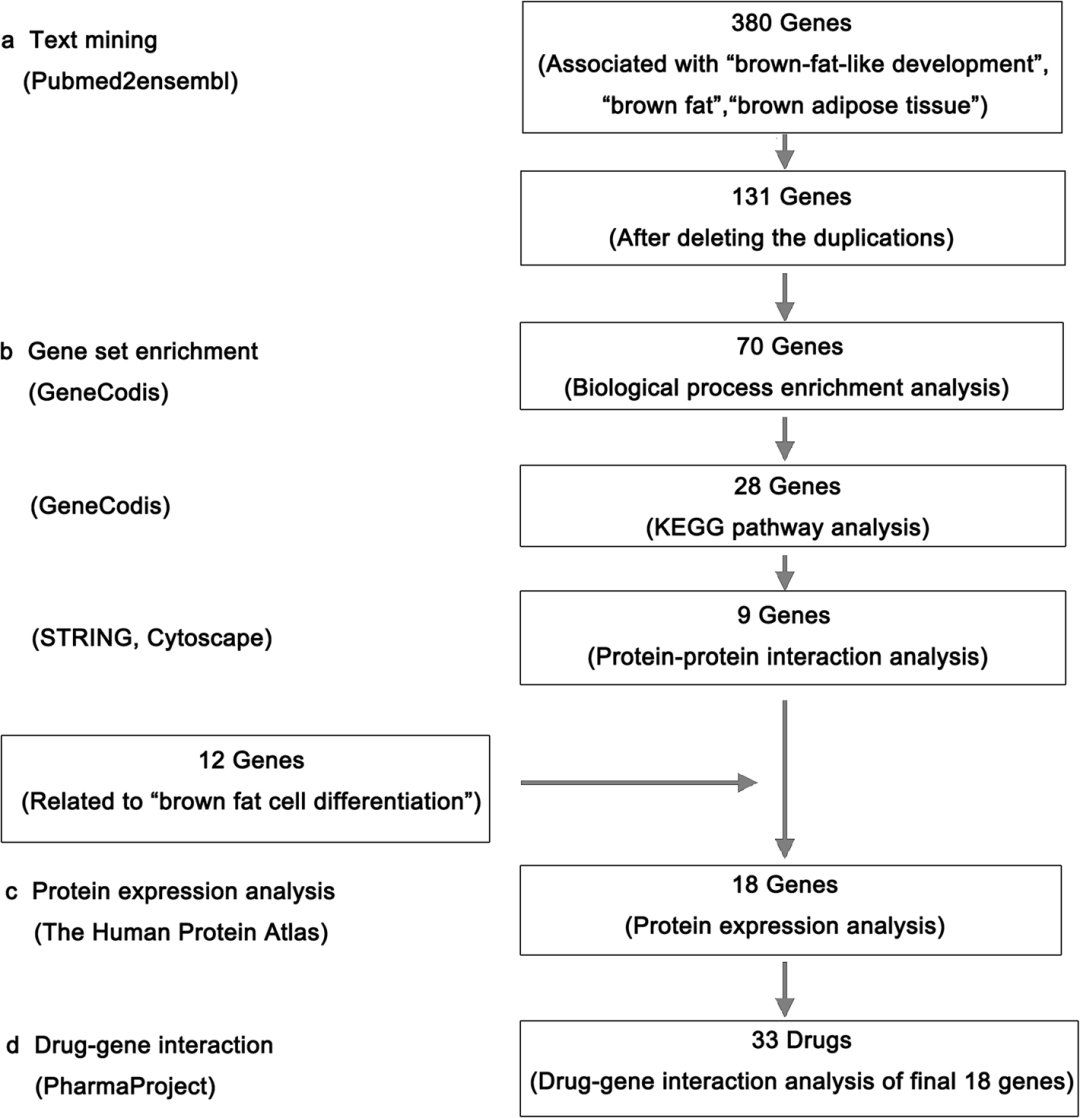
Summary of data mining results. **a** Text mining: Text mining was performed by using the terms “brown-fat-like development”, “brown fat”, and “brown adipose tissue”, respectively. One hundred seventy-eight genes were found by using pubmed2ensembl and total of 121 genes were common to all the three lists. **b** Gene set enrichment: Biological process and pathway analysis were performed in GeneCodis to enrich 70 and 28 genes, respectively. Next, nine significant genes were derived by protein-protein interaction analysis using STRING and Cytoscape. Together with the 12 genes from the most enriched biological process, “brown fat cell differentiation”, 18 significant genes were selected for the final analysis. **c** Protein expression analysis: Protein expression analysis was performed using The Human Protein Atlas. **d** Drug-gene interactions: 33 drugs were selected targeting white fat browning, which may serve as potential treatments of obesity and related metabolic diseases

According to the data mining strategy described in Fig. 1, in the text mining process, 380 genes were found related to brown-fat-like development, brown fat, and BAT and 131 genes were common to all the three lists (Fig. 2). The GO biological process annotations performed in GeneCodis showed that the most highly enriched terms were closely related to the brown-fat-like pathology. We selected a *P* value (*P* = 1.00e−07) as the cutoff, because a *P* value cutoff (*P* = 1.00e−08) would exclude biological processes which contribute significantly to the process of brown-fat-like development, such as “cellular lipid metabolic process”, while the selection of *P* value (*P* = 1.00e−06) would include biological processes irrelevant to the process of brown-fat-like development, which would affect the precision and accuracy of the results. Therefore, to ensure that only the most enriched biological processes relevant to brown-fat-like development were selected, a *P* value (*P* = 1.00e−07) was chosen as the cutoff, which resulted in 16 sets of annotations containing 70 genes (Table 1). The three most enriched biological process annotations were (1) “brown fat cell differentiation” (*P* = 8.31e−21), (2) “response to cold” (*P* = 7.85e−16), and (3) “positive regulation of transcription from RNA polymerase II promoter” (*P* = 2.92e−12), including 12, 10, and 20 genes, respectively, among which the lower *P* value indicates that the biological process is more relevant to brown-fat-like development. Other highly enriched biological process annotations included “lipid biosynthetic process”, “cellular lipid metabolic process”, “glucose homeostasis”, and “response to insulin stimulus”. In the analysis of enriched KEGG pathway annotations, the *P* value cutoff was set to *P* = 1.00e−06, which resulted in 28 genes in six pathways above the cutoff (Table 2). These enriched KEGG pathways annotations were (1) “PPAR signaling pathway” (*P* = 1.28e−14), (2) “adipocytokine signaling pathway” (*P* = 3.87e−13), (3) “pathways in cancer” (*P* = 1.10e−09), (4) “insulin signaling pathway” (*P* = 4.39e−09), (5) “prostate cancer” (*P* = 1.89e−07), and (6) “type II diabetes mellitus” (*P* = 2.15e−07).

**Table 1:** Summary of biological process gene set enrichment analysis

Progress	Genes in query set	Total genes in genome	Corrected hypergeometric *P* value	Genes
Brown fat cell differentiation	12	26	8.3154e−21	*PPARG*, *PIK3R1*, *MRAP*, *PPARGC1A*, *SLC2A4*, *ADIG*, *ADIPOQ*, *ADRB1*, *PRDM16*, *ADRB3*, *CEBPB*, *FABP4*, *UCP1*
Response to cold	10	29	7.84828e−16	*PPARG*, *ACOT11*, *PMCH*, *AGT*, *ADRB1*, *UCP3*, *GMPR*, *ADRB3*, *TRH*, *IL6*
Positive regulation of transcription from RNA polymerase II promoter	20	578	2.90244e−12	*NFKB1*, *PPARG*, *NFE2L2*, *TWIST1*, *SREBF1*, *PPARA*, *PIK3R1*, *LPIN2*, *ESR1*, *CTNNB1*, *HNF4A*, *IL10*, *RXRA*, *EN1*, *TGFB1*, *ESRRA*, *IL6*, *CREBBP*, *PPARGC1B*
Lipid biosynthetic process	8	29	7.45936e−12	*SREBF1*, *DGAT2L6*, *MOGAT2*, *DGAT2*, *PRKAA2*, *MOGAT3*, *MOGAT1*, *AWAT1*
Cellular lipid metabolic process	11	128	6.70379e−11	*CYP7A1*, *LPIN1*, *PPARA*, *DGAT2*, *PRKAA2*, *LPIN2*, *AGT*, *DGAT1*, *RXRA*, *CREBBP*, *UCP1*
Glucose homeostasis	9	64	7.26406e−11	*PPARG*, *SLC2A4*, *PMCH*, *IRS1*, *PRKAA2*, *DBH*, *HNF4A*, *ADIPOQ*, *INS*
Response to drug	14	301	1.68321e−10	*PPARG*, *FXN*, *SREBF1*, *PIK3R1*, *CTNNB1*, *HNF4A*, *IL10*, *LPL*, *CYP19A1*, *TGFB1*, *NOS3*, *IL6*, *LIPE*, *CFTR*
Lipid metabolic process	13	241	1.74841e−10	*FADS2*, *PPARG*, *SREBF1*, *PPARA*, *LPIN2*, *LEP*, *LPL*, *UCP3*, *CIDEA*, *APOC3*, *LPIN3*, *FABP4*, *LIPE*
Triglyceride biosynthetic process	7	33	7.84617e−10	*LPN1*, *MOGAT2*, *DGAT2*, *LPIN2*, *LPL*, *DGAT1*, *GPAT2*
Response to hypoxia	11	175	9.5641e−10	*PPARA*, *LEP*, *NRF1*, *MMP2*, *UCP2*, *UCP3*, *TRH*, *TGFB1*, *ADIPOQ*, *NOS3*, *CREBBP*
Positive regulation of MAPK cascade	8	61	1.16264e−09	*LEP*, *CTNNB1*, *AGT*, *IGF1R*, *CNTF*, *ADRB3*, *IL6*, *INS*
Response to insulin stimulus	8	62	1.2526e−09	*MC4R*, *RETN*, *PPARA*, *IRS1*, *LEP*, *IL10*, *UCP3*, *IL6*
Aging	9	114	5.88491e−09	*SREBF1*, *AGT*, *ADRB1*, *UCP3*, *ADRB3*, *RXRA*, *TGFB1*, *NOS3*, *IL6*
Response to estradiol stimulus	8	89	1.57804e−09	*PIK3R1*, *ESR1*, *DBH*, *NRF1*, *CTNNB1*, *CYP19A1*, *TGFB1*, *NOS3*
Cellular lipid metabolic process	7	56	1.62076e−08	*PPARG*, *LPIN1*, *SLC2A4*, *IRS1*, *UCP2*, *RXRA*, *ADIPOQ*
Response to organic cyclic compound	8	112	8.05856e−08	*FXN*, *STC1*, *DBH*, *CYP19A1*, *RXRA*, *TRH*, *NOS3*, *IL6*

Among the most significantly enriched biological processes above the cutoff, those most relevant to brown-fat-like development based on the available literature and research were selected. The analysis of enriched biological process annotations resulted in 16 sets of annotations containing 70 genes

**Table 2:** Summary of KEGG process gene set enrichment analysis

Progress	Genes in query set	Total genes in genome	Corrected hypergeometric *P* value	Genes
PPAR signaling pathway	10	70	1.27872e−14	*CYP7A1*, *FADS2*, *FABP4*, *PPARA*, *ADIPOQ*, *UCP1*, *LPL*, *RXRA*, *PPARG*, *APOC3*
Adipocytokine signaling pathway	9	67	3.87352e−13	*IRS1*, *SLC2A4*, *PPARA*, *ADIPOQ*, *NFKB1*, *LEP*, *PRKAA2*, *RXRA*, *PPARGC1A*
Pathways in cancer	11	324	1.10046e−09	*IL6*, *MMP2*, *IGF1R*, *FGF19*, *NFKB1*, *PIK3R1*, *TGFB1*, *CTNNB1*, *CREBBP*, *RXRA*, *PPARG*
Insulin signaling pathway	8	133	4.39226e−09	*IRS1*, *LIPE*, *SLC2A4*, *SREBF1*, *PIK3R1*, *PRKAA2*, *INS*, *PPARGC1A*
Prostate cancer	6	88	1.89023e−07	*IGF1R*, *NFKB1*, *PIK3R1*, *INS*, *CTNNB1*, *CREBBP*
Type II diabetes mellitus	5	46	2.15133e−07	*IRS1*, *SLC2A4*, *ADIPOQ*, *PIK3R1*, *INS*

Among the most significantly enriched pathway annotations above the cutoff, those most relevant to brown-fat-like development based on the available literature and research were selected. The analysis of enriched pathway annotations resulted in six pathways containing a total of 28 unique genes

### Results of protein-protein interaction

**Fig. 3:**
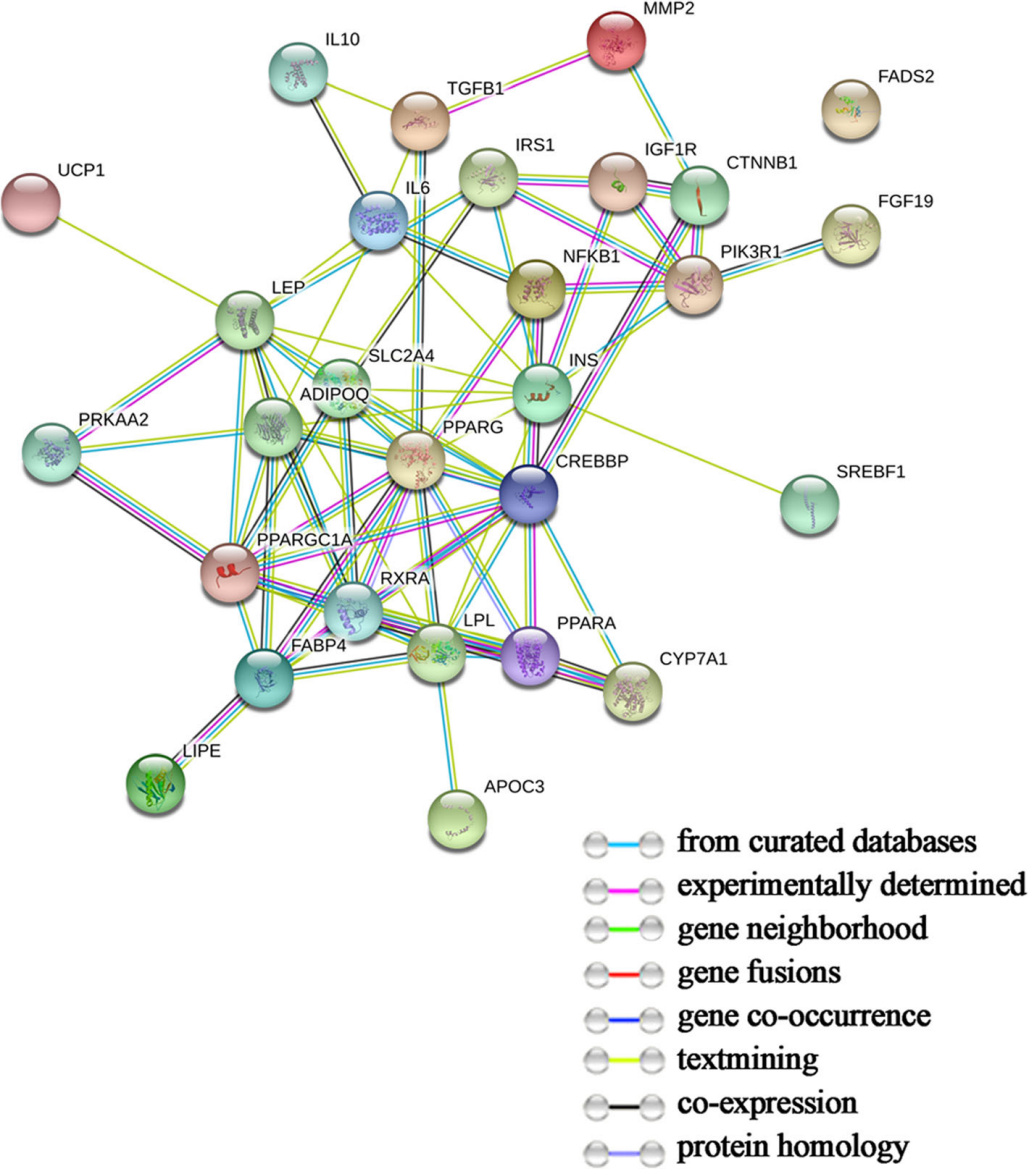
The protein-protein high (confidence score 0.900) interaction network of the 28 targeted genes using STRING. Network nodes represent proteins and different colored edges represent protein-protein interactions

**Fig. 4:**
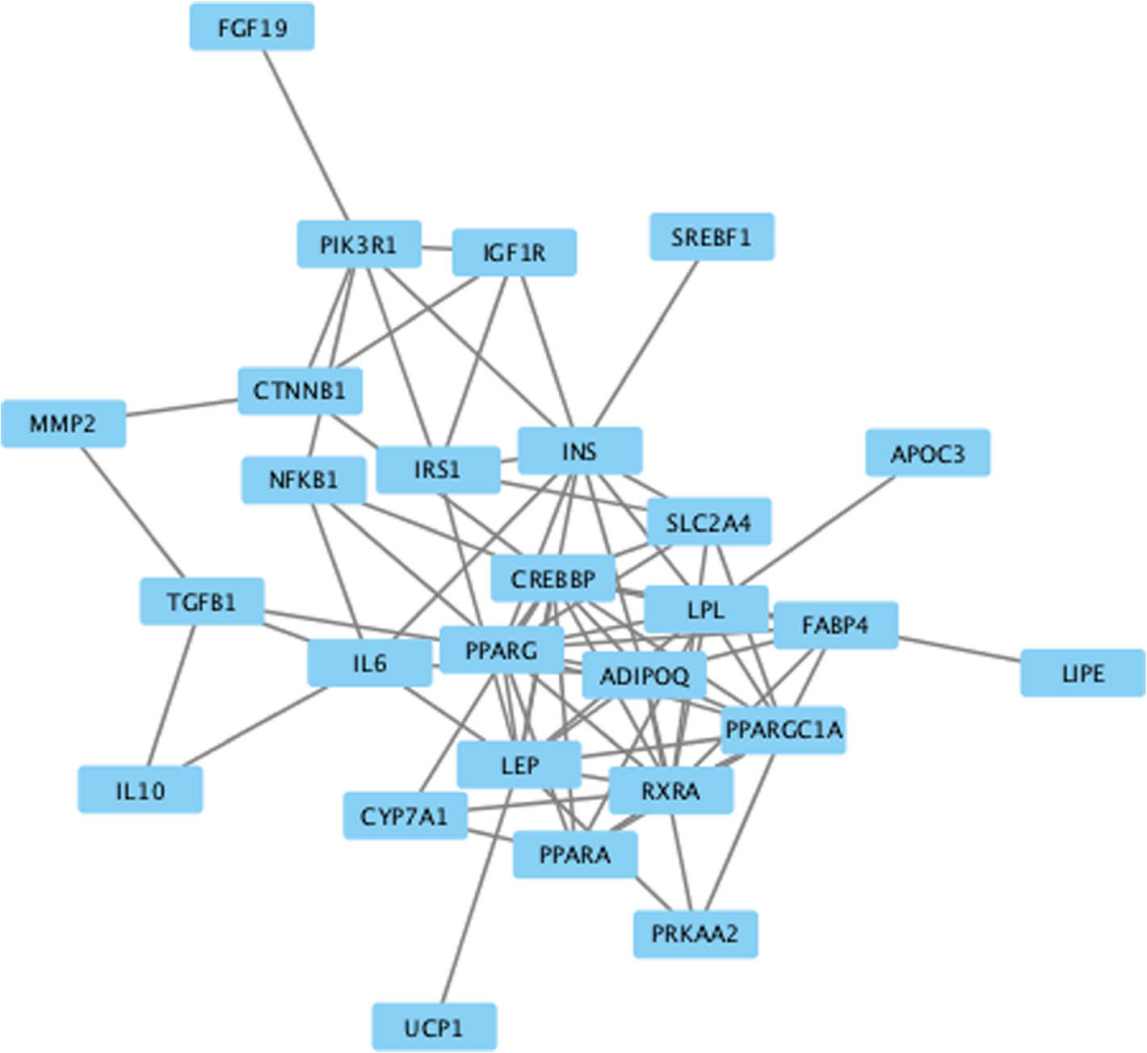
The protein-protein interaction network of the 27 targeted genes by using Cytoscape. Network nodes represent proteins and edges represent protein-protein interactions

The protein-protein interaction analysis was performed in STRING (Fig. 3). One of the target genes, *FADS2*, was found isolated from other nodes, which means there is no interaction between *FADS2* and other genes. The visualization of the network of 27 genes yielded from STRING was demonstrated in Cytoscape (Fig. 4). CentiScaPe was then used to enumerate and analyze the topological characteristics of each node. “Degree”, as an important parameter, stands for the total number of edge incident to the node, while betweenness centrality for each node represents the number of shortest paths that pass through the node. The average values of degree and betweenness in this study were 32.00 and 5.56, respectively. According to the criterion that the nodes for which degree and betweenness were both greater than or equal to the mean were the key nodes, nine genes were derived, including “*PPARG*”, “*CREBBP*”, “*LEP*”, “*INS*”, “*ADIPOQ*”, “*LPL*”, “*FABP4*”, “*IL6*”, and “*PIK3R1*”.

### Results of protein expression analysis

**Fig. 5:**
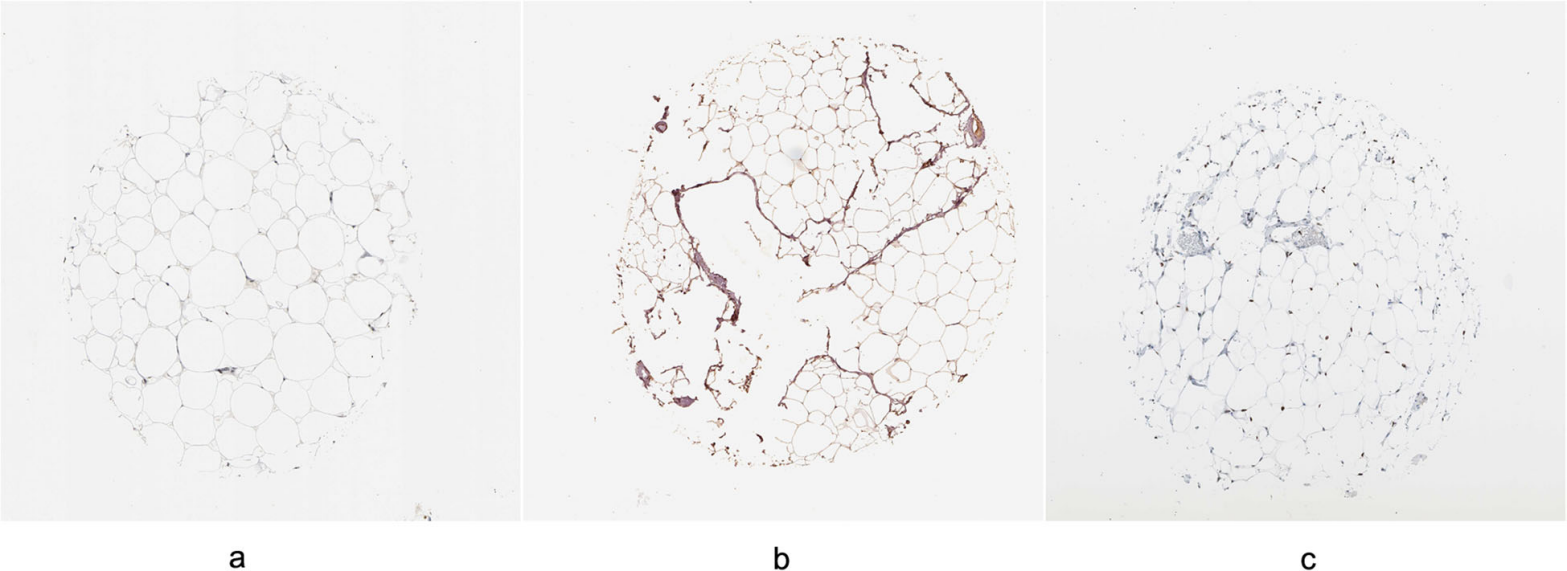
The protein expression analysis by using the Human Protein Atlas. **a** PPARG was positively expressed in adipose tissue. **b** ADRB3 was positively expressed in adipose tissue. **c** CEBPB was positively expressed in adipose tissue

We used the Human Protein Atlas to generate tissue-based immunohistochemically stained images of the expression of key proteins, PPARG (peroxisome proliferator-activated receptor gamma), ADRB3 (adrenoceptor beta 3), and CEBPB (CCAAT/enhancer-binding protein beta). These proteins were positively expressed in adipose tissue (Fig. 5).

### Results of drug-gene interactions

Considering the fact that the most enriched biological process, “brown fat cell differentiation”, contributes significantly to the process of brown-fat-like development and the genes related to this process may serve as the potential targets, we supplemented the 12 genes from the query set of “brown fat cell differentiation” to the final list. After deleting the duplications, 18 genes made up the final list, including “*PPARG*”, “*CREBBP*”, “*LEP*”, “*INS*”, “*ADIPOQ*”, “*LPL*”, “*FABP4*”, “*IL6*”, “*PIK3R1*”, “*MRAP*”, “*PPARGC1A*”, “*SLC2A4*”, “*ADIG*”, “*ADRB1*”, “*PRDM16*”, “*ADRB3*”, “*CEBPB*”, and “*UCP1*”. Using these 18 genes as potential targets in drug-gene interaction analysis, a list of 622 drugs was initially obtained. After filtering those by the status of “launched”, “phase I/II/III clinical trial”, “pre-registration” or “registered”, a list of 158 drugs was selected. We took a close look at the details of each drug, especially the “Trialtrove Trials”, excluded those drugs that were not detailed in the development process, and got 81 drugs. Notably, considering the efficacy and safety of the drugs and their possible potential hazards to other systems, drugs including beta 1 adrenoceptor antagonists and agonists, interleukin-6 antagonists, and CREB-binding protein inhibitors were removed from the drug lists. Finally, a list of 33 drugs was selected as a possible drug treatment for brown-fat-like development. According to the therapeutic targets, including *PPARG*, *ADRB1*, *ADRB3*, *SLC2A4*, *INS*, and *LPL*, these drugs can be divided into PPAR-γ agonists, β3-AR agonists, insulin sensitizer, insulin, lipase clearing factor stimulant, and other drugs.

Among these drugs, four drugs targeting *ADRB3* are selected as possible therapeutic agents for the treatment of obesity, consistent with the biological process of “brown fat cell differentiation”. In physiological conditions, cold stimulation activates BAT by releasing adrenaline through sympathetic nerves, increasing the expression of genes involved in heat production and lipid metabolism, promoting the accumulation of beige fat [16]. Agonists for β3-AR, including vibegron, mirabegron, and solabegron, are currently pursued for clinical use in overactive bladder (OAB) and urinary incontinence. A study showed that mirabegron led to higher human BAT metabolic activity as measured by 18F-flurordeoxyglucose (18F-FDG) PET/CT and increased resting metabolic rate, which might be a potential treatment for obesity and diabetes [10]. The other two drugs, solabegron which is being pursued for irritable bowel syndrome and vibegron which received approval in 2018 for the treatment of OAB, have not been studied for effects on obesity and metabolic diseases so far [17].

Eighteen of the drugs in the final drug list were PPAR-γ agonists, which is in accordance with the most enriched pathway “PPAR signaling pathway”. Among these, several drugs belonging to the thiazolidinedione (TZD) class are used in the treatment of type 2 diabetes. Pioglitazone is used as an adjunct to diet, exercise, and other diabetes medications (e.g., glimepiride, metformin, and alogliptin) to manage type 2 diabetes by stimulating the nuclear receptor PPAR-γ, promoting insulin sensitivity and improving the uptake of blood glucose [18, 19]. Other drugs of the TZD class, rosiglitazone and lobeglitazone, whose latter is a dual PPAR-α/γ agonist, work as insulin sensitizers by binding to the PPAR in adipocytes as a stand-alone drug or in combination with metformin or other drugs [20, 21]. Distinct from the TZD class of PPAR-γ full agonists, INT131 shows strong hypoglycemic activity in rodent models of diabetes with reduced side effects [22]. Chiglitazar, a dual PPAR-α/γ agonist, is an effective insulin sensitivity agent used to treat the major metabolic disorders of type 2 diabetes and may bring better impacts on lipid homeostasis through different mechanisms than selective PPAR-γ agonists in diabetic patients [23]. Another dual agonist, saroglitazar, is indicated for diabetic dyslipidemia and hypertriglyceridemia when type 2 diabetes cannot be controlled by statin therapy [24]. In addition to treating diabetes, other PPAR-γ agonists in the list are currently under the phase I/II clinical trial for the treatment of hyperuricemia (arhalofenate), non-alcoholic steatohepatitis (lanifibranor and T3D-959), systemic sclerosis (lanifibranor), Alzheimer’s disease (T3D-959), and multiple sclerosis (VCE-004.8, [25–28]).

Among the list, seven drugs work as lipase clearing factor stimulants targeting *LPL* which is present in “PPAR signaling pathway”, “lipid metabolic process”, and “triglyceride biosynthetic process”. The activation of BAT and beige adipocytes is accompanied by a significant increase in intravascular LPL activity, driving FA into thermogenic adipocytes. Accordingly, the activation of BAT may lower plasma TG and obesity [29]. Among these drugs, three fibrates including bezafibrate, ciprofibrate, and etofibrate are used as lipid-lowering agents as well as PPAR agonists to treat hypercholesterolemia, hypertriglyceridemia, and reduce insulin resistance when the dyslipidemia is associated with other metabolic syndromes, for example, type 2 diabetes [30, 31]. Other drugs included insulin used for diabetes, vanadium + aonys in the clinical trial for type 2 diabetes, and alimentary/metabolic disease, targeting *INS* and *SLC2A4*, respectively. Vanadium, a trace metal, plays a crucial role in maintaining blood glucose homeostasis possibly through its insulin-mimetic effects [32]. Noticeably, one drug in our drug list, metoprolol + tesofensine, is currently in the phase II clinical trial for the treatment of obesity. Tesofensine, a serotonin-noradrenaline-dopamine reuptake inhibitor, was originally investigated for the treatment of Alzheimer’s disease and Parkinson’s disease and was found to be effective in weight loss in obese patients by suppressing appetite and increasing resting energy expenditure [33, 34].

## Discussion

Obesity is becoming one of the greatest public health epidemics in the twenty-first century, with approximately two billion adults worldwide currently classified as overweight or obese which is related to many metabolic diseases [3]. However, there is currently no ideal drug to treat obesity. Given its potential role in stimulating energy expenditure, the discovery of active BAT in adult humans raised the expectations for the development of new anti-obesity treatments [8, 35]. Key regulators in BAT differentiation play an important role in the process of white fat browning and activation of BAT, and these sites are expected to become new targets for drug therapy for obesity. In this study, we identified 18 genes that may regulate the process of brown fat-like development via gene set enrichment analysis and a list of 35 drugs was compiled targeting these key genes (Additional file 1). A variety of mechanisms associated with brown and beige fat activation and development have been proposed so far, which is consistent with our findings of the enriched biological processes (Table 1), such as “response to cold”, “lipid biosynthetic process”, “cellular lipid metabolic process”, “glucose homeostasis”, “positive regulation of MAPK cascade”, and “response to insulin stimulus”. Potential drugs can be divided into PPAR-γ agonist, β3-AR agonist, insulin sensitizer, insulin, and lipase clearing factor stimulant, acting on signaling pathway including “PPAR signaling pathway”, “adipocytokine signaling pathway”, “insulin signaling pathway”, and “type II diabetes mellitus” (Table 2).

BAT is a specialized tissue responsible for heat production, more specifically for adaptive non-shivering thermogenesis that corresponds to adaptation to cold environments or diet modifications [8]. Cold exposure has an important regulatory effect on the activation of BAT and browning of WAT, which has been reported in both mice and humans [36, 37]. UCP1, the hallmark of brown adipocytes, is responsible for thermogenesis through uncoupling the proton leakage from the ATP production [38]. Cold exposure to BAT is mainly induced by β3-AR-cyclic adenosine monophosphate (cAMP)-protein kinase A (PKA) signaling pathway, which induces UCP1 expression in BAT. In response to cold, norepinephrine (NE) is released by the sympathetic nervous system and binds to the β3-AR on the membrane of brown and beige adipocytes, which results in the activation of adenylyl cyclase to produce cAMP that activates PKA [39]. PKA phosphorylates and activates cAMP response element-binding protein (CREB) resulting in enhanced transcription of UCP1 and PPAR-γ co-activator (PGC-1α) [40]. In addition, the activation of PKA enhances the phosphorylation of hormone-sensitive lipase (HSL) and promotes the decomposition of TG in lipid droplets into glycerol and FA [39]. FA then enters the mitochondria for the citric acid cycle, resulting in the activation of the electron transport chain and uncoupled respiration through UCP1, which results in the generation of heat instead of ATP [41]. At the same time, the adipose tissue is also an endocrine organ, which can secrete a variety of adipocytokines, such as leptin and adiponectin, and regulate the sensitivity of insulin and energy metabolism. Recent studies have found that BAT can also produce some secretory factors, such as neuregulin 4 (NRG4), fibroblast growth factor 21 (FGF21), vascular endothelial growth factor (VEGF), bone morphogenetic protein (BMP), interleukin-6 (IL-6), adiponectin, and other factors, to maintain metabolic balance in the body [42–44].

Agonist-mediated activation of β3-AR is considered as a targeted therapeutic strategy. Previous phase II clinical trials with β3-AR agonists demonstrated improved glucose tolerance, increased FA oxidation, and resting metabolic rate, but none of them eventually passed the trials and achieved the approval for the treatment of obesity [45–48]. Limiting factors included poor oral bioavailability and undesirable activation of the cardiovascular system from off-target binding to the β1-AR [49, 50]. Recently, a new β3-AR agonist, mirabegron, was approved to treat OAB at a clinically indicated dose of 25 or 50 mg/day [51]. Given its satisfactory bioavailability as well as a higher in vitro binding affinity for the human β3-AR, mirabegron is considered as a promising candidate [52]. In a recent study of the β3-AR agonist mirabegron, 12 healthy male volunteers were randomly given one-time doses of placebo, 50 mg (i.e., the approved dose) and 200 mg of the drug, respectively [53]. Only those in the 200-mg dose group showed both increased nonesterified FAs (+ 68%) and resting energy expenditure (+ 5.8%); these data suggest the clinical feasibility of stimulating BAT thermogenesis and WAT lipolysis. Moreover, chronic mirabegron treatment (10 weeks, 50 mg/day) has been found to induce UCP1, transmembrane protein-26 (TMEM26), cell death-inducing DNA fragmentation factor-α-like effector A (CIDEA), and phosphorylation of HSL on serine-660 in obese subjects; these findings indicate that long-term mirabegron administration might help in converting BAT to WAT [54]. To summarize briefly, given the limiting factors such as oral bioavailability and off-target β1-AR-mediated cardiovascular effects, using novel β3-AR agonists to treat obesity seems promising, but will require long-term trials to confirm their efficacy, safety, and ability to be tolerated.

**Fig. 6:**
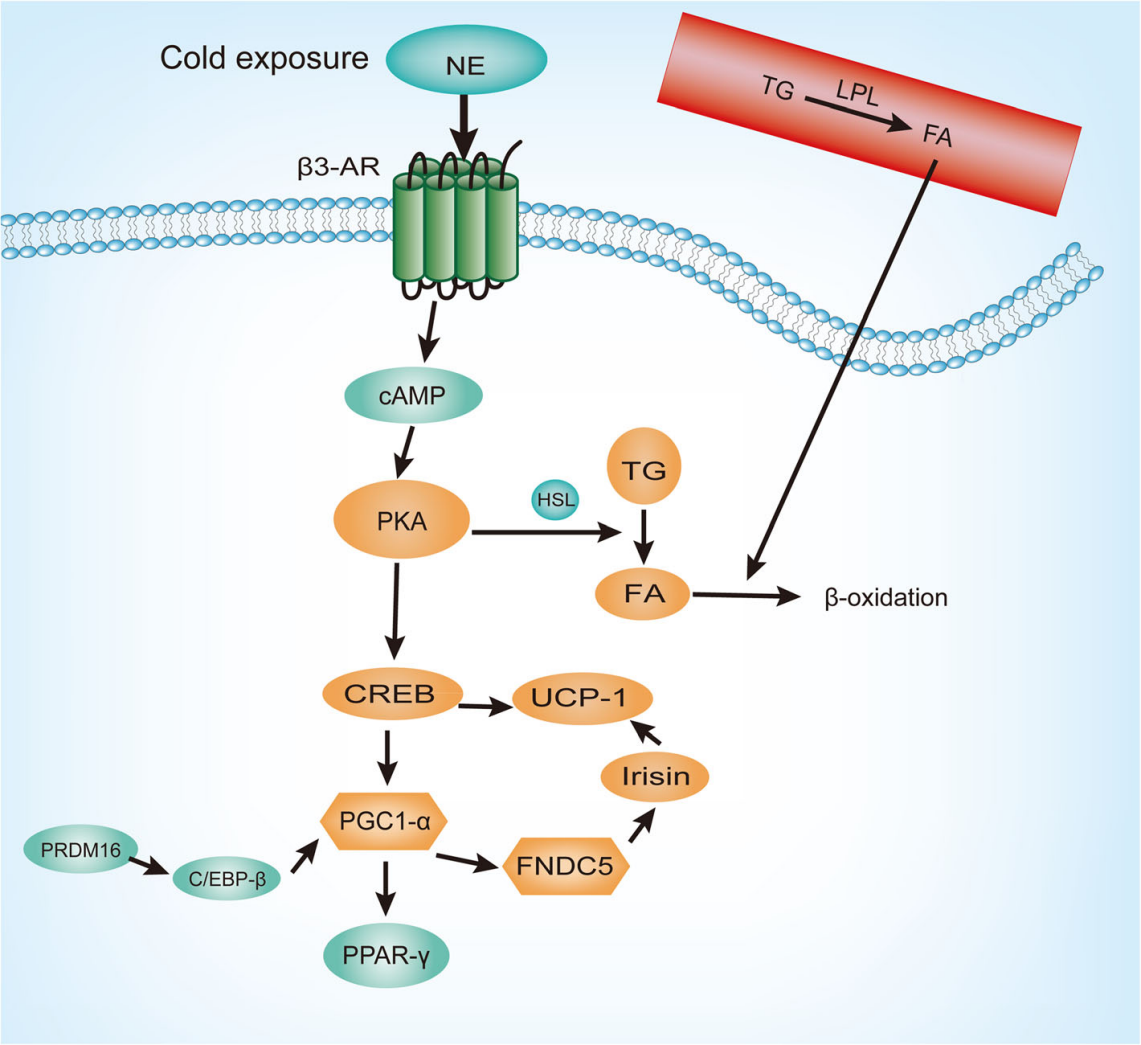
Key genes and signaling pathways involved in the brown fat activation and white fat browning**.** In response to cold, norepinephrine (NE) is released by the sympathetic nervous system and binds to the β3-adrenergic receptor (β3-AR) on the membrane of brown and beige adipocytes, which results in the activation of adenylyl cyclase to produce cyclic adenosine monophosphate (cAMP) that activates protein kinase A (PKA). PKA phosphorylates and activates cAMP response element-binding protein (CREB) resulting in enhanced transcription of uncoupling protein-1 (UCP1) and PPAR-γ co-activator (PGC-1α). Activated PGC1-α also stimulates the expression of fibronectin type III domain-containing protein 5 (FNDC5) which encodes a type I membrane protein that is cleaved to form a newly identified hormone, irisin. Irisin works on white adipocyte to stimulate UCP1 expression. Another factor, positive regulatory domain containing 16 (PRDM16), forms a transcriptional complex with CCAAT enhancer-binding protein (C/EBP)-β and induces the expression of PPAR-γ and PGC1-α. In addition, the activation of PKA enhances the phosphorylation of hormone-sensitive lipase (HSL) and promotes the decomposition of triacylglycerol (TG) in lipid droplets into glycerol and fatty acids (FA) that enter the mitochondria for β-oxidation. Activated BAT replenishes these stores via the uptake of TG-derived FA, generated by lipoprotein lipase (LPL)-mediated hydrolysis of TG

When β3-AR/cAMP/PKA signaling is activated, the key downstream activation of p38 mitogen-activated protein kinase (MAPK) stimulates the activating transcription factor 2 (ATF-2), driving PGC1-α transcription [55]. PGC1-α is a co-activator of PPAR-γ that upregulates gene expression of UCP1 and activates PPAR, mitochondrial biogenesis, and oxidative metabolism. Studies have shown that activated PGC1-α also stimulates the expression of fibronectin type III domain-containing protein 5 (FNDC5) encoding a type I membrane protein that is cleaved to form a hormone, irisin. Irisin works on white adipocyte in culture and in vivo to stimulate UCP1 expression and the process of brown-fat-like development [56]. Another factor, positive regulatory domain containing 16 (PRDM16), is the decisive regulator of the differentiation of preadipocytes into brown adipocytes, by forming a transcriptional complex with CCAAT enhancer-binding protein (C/EBP)-β and inducing the expression of PPAR-γ and PGC1-α [57, 58] (Fig. 6). The regulatory factors involved in the above processes and pathways were consistent with the key genes of our findings, such as *PPARG*, *INS*, *PPARGC1A*, *PRDM16*, *ADRB3*, *CEBPB*, and *UCP1*.

PPARs are a family of nuclear transcription factors that is very important in the regulation of energy metabolism, including lipids, adipogenesis, and glucose control. Among the three subtypes of PPARs, α, β/δ, and γ, PPAR-α is a major activator of FA oxidation pathways and is the target of the hypolipidemic fibrate drugs, expressed mainly in high-energy requiring tissues such as the skeletal muscles, liver, heart, and BAT, while PPAR-γ is predominantly expressed in WAT and BAT, where it is a major regulator of adipogenesis as well as a whole-body lipid metabolism and insulin sensitivity [59]. Activation of PPAR-γ by the TZD class of drugs has been pursued for type 2 diabetes and has been considered as a therapeutic strategy for the treatment of obesity. The full PPAR-γ agonists of TZD, such as rosiglitazone, have shown binding and stabilizing helix12 of the PPAR-γ ligand binding domain (LBD), resulting in extended accumulation of PRDM16 [60]. However, since the 292–338 region of PGC1-α is a ligand-independent PPAR-γ binding region, which is also involved in fat synthesis and the activation of PPAR-γ leading to accumulation of TG, which shows side effects such as weight gain. In addition, meta-analyses of clinical trials have implicated rosiglitazone in increasing the risk of cardiovascular disease but not pioglitazone, which may be interpreted in part by pioglitazone having more off-target effects, such as agonist of PPAR-α. In this regard, dual or pan PPAR agonists are considered as a promising treatment that preserves the strong efficacy and eliminates undesired side effects.

Fibrate drugs (e.g., bezafibrate, ciprofibrate, etofibrate, and gemfibrozil) are also potent activators of PPAR-α, which are used in hypercholesterolemia primarily due to their ability to substantially increase HDL levels and lower TG levels. In humans, the activation of PPAR-α by fibrate has shown to increase the circulating levels of antiatherosclerotic HDL-cholesterol that lowers TG levels, liberates FA and apolipoprotein CIII (apo-CIII), improves the overall atherogenic plasma lipid profile, and has beneficial effects on inflammation, insulin resistance, and metabolic syndrome. Fibrates also improve HDL and TG levels via induction of apo-AI, apo-AII, and apo-AV, respectively [61]. As shown in researches, the activation of BAT plays a key role in lipoprotein metabolism and atherosclerosis. More specifically, when PKA phosphorylates and activates UCP1 and PGC-1α, it also promotes the activity of lipases associated with the intracellular lipid droplets, leading to the release of FA that enters the mitochondria for β-oxidation. Activated BAT replenishes these stores via the uptake of TG-derived FA, generated by LPL-mediated hydrolysis of TG. As a result, BAT activation lowers down plasma TG and cholesterol levels [40] (Fig. 6).

Unexpectedly, one combination medication, metoprolol + tesofensine, is on our final drug list based on the drug-gene interaction, the former of which targets ADRB1. Tesofensine is a presynaptic reuptake inhibitor of serotonin, norepinephrine, and dopamine [62]. According to a phase II study, after given placebo, 0.25 mg, 0.5 mg, or 1.0 mg/day orally, respectively, along with an energy-restricted diet for 24 weeks, the mean weight loss of 101 obese and nondiabetic subjects was 4.5%, 9.2%, and 10.6% of initial body weight. Positive effects on blood TG, insulin, cholesterol, and hemoglobin A1c (HbA1c) were also observed [63]. Studies have found that tesofensine induces weight loss primarily through the reduction of food intake with a small increase in metabolic rate [64]. In addition, the upregulation of dopaminergic pathways partially contributes to the reduction in food intake due to blockade of presynaptic reuptake [65].

The limitations of this study stem from the databases we used and the criteria we set in each screening step. Thus, our analysis may have to be repeated when databases become more comprehensive. Additionally, different rational criteria could be set up to further explore the best outcome.

## Conclusions

In conclusion, we presented a method to explore potential drugs that target the pathways that are relevant to BAT or the browning of WAT to ameliorate obesity and associated comorbidities. Such methods may be used at intervals when databases and analytical tools evolve and improve. As a result, with the method presented, we identified a total of 33 potential drugs, including 18 PPAR-γ agonists, 4 β3-AR agonists, 1 insulin sensitizer, 3 insulins, 6 lipase clearing factor stimulants, and other drugs. Our list of candidate drugs participates and regulates the process of the activation of BAT or white fat browning through different pathways and mechanisms, which provides a basis for the development of novel targeted therapies as potential treatments for obesity and related metabolic diseases.

## Methods

### Text mining

Text mining is the process of deriving genomic data from published biomedical literatures. In this study, we used pubmed2ensembl [66] to perform text mining. pubmed2ensembl is a database based on an extended version of the BioMart system which links over two million articles in PubMed to approximately 150,000 genes in Ensembl from 50 different species [67]. A list of genes associated with a particular biological phenomenon can be generated in the process of biological exploration in pubmed2ensembl. In this study, we determined the three queries with the terms “brown-fat-like development”, “brown fat”, and “brown adipose tissue”, respectively, in the species dataset of “*Homo sapiens*”. We selected “Ensembl Gene ID” and “Associated Gene Name” under GENE and deselected “MEDLINE: PubMed ID” under PUBMED2ENSEMBL FEATURES. “Search for PubMed IDs” and “filter on Entrez: PMID” drop-down menus were chosen in the search of every query. Then, the queries returned three lists of genes, the intersection of which was then used as the starting point for the next steps.

### Biological process and pathway analysis

We used GeneCodis [68] for an enrichment analysis of the genes related to brown-fat-like development, brown fat, and BAT intersection. GeneCodis is a web-based tool for finding biological annotations that frequently co-occur in a set of genes from different sources (for example, KEGG pathways, GO, Swiss-Prot keywords, and InterPro motifs) and rank them by statistical significance [69, 70]. We input the genes from the text mining step and analyzed the annotations in regard to the biological process involved. The most significantly enriched biological processes were selected. Genes involved in the selected annotations were then performed with an additional GeneCodis analysis, that is, annotations of the Kyoto Encyclopedia of Genes and Genomes (KEGG) pathways. Genes in the most highly enriched pathways were used for further analysis.

### Protein interaction network

The STRING [71] was used to analyze the protein-protein interactions of the selected genes [72]. We input the genes from the previous step and selected “*Homo sapiens*” as the organism. To screen the genes with a strong interaction, the confidence score was set to the highest score (0.900) in this study. Then the protein-protein interaction network of the target genes was obtained.

To further narrow the candidate gene field, we used the Cytoscape (The Cytoscape Consortium, USA) software to analyze the interaction network. Cytoscape is a software platform for visualizing, analyzing, and interpreting biomedical interaction networks supported by diverse annotations, gene expression profiles, and experimental data [73]. We imported data from the previous STRING EXPORT channel. In order to select the key nodes, an app, CentiScaPe (The Cytoscape Consortium, USA), enumerating a number of network parameters, was used to analyze the topological characteristics of each node. “Degree” and “betweenness” as parameters were chosen to select the key genes. The “degree” value of a node means the number of gene products that interact with each other, which indicates the role that the node plays in the browning of WAT, while the “betweenness” value indicates the tendency that a node connects to the core of other nodes. In this study, a criterion that “degree” and “betweenness” were both greater than or equal to the mean was the key nodes that were set. Candidate genes were also selected for further analysis.

### Protein expression analysis

The Human Protein Atlas [74] is a freely available database which provides a map of the human tissue proteome on a tissue and organ level combined with protein profiling using immunohistochemistry based on 24,028 anti-bodies corresponding to 16,975 protein-encoding genes [75]. We input the key genes selected from the previous steps and obtained immunohistochemically stained images of protein expressions in specific tissues and organs (e.g., adipose tissue, and the liver) to explore and analyze the expression of a certain protein on a tissue and organ level.

### Drug-gene interactions

Pharmaprojects [76] is an online database covering pharma R&D across global markets with over 68,000 drug profiles including 15,000 drugs in active development and supporting fast access to drug development results for specific diseases, companies, and countries [77]. The final list of genes was used as the potential targets in a search for existing drugs. We input the final list of genes and chose the targets in accordance with the genes. Then the query returned all the drug hits and provided the global status, drug disease, therapeutic class, mechanism of action, target, and other information on each drug. The candidate drugs were selected by screening the global status of “launched”, “phase I/II/III clinical trial”, “pre-registration”, or “registered”, for these stages are indicative of relative mature phases of pharmaceutical development. The final drug list was obtained and may represent the potential treatments relevant to white fat browning.

## Supplementary information

**Additional file 1.** Candidate drugs targeting genes associated with brown fat or white fat browning. (XLSX 24 kb)

## Data Availability

The datasets supporting the conclusions of this article are available at pubmed2ensembl (http://pubmed2ensembl.ls.manchester.ac.uk/), GeneCodis (http://genecodis.cnb.csic.es/), and Pharmaprojects (https://pharmaintelligence.informa.com/).

## Abbreviations

18F-FDG: 18F-flurordeoxyglucose
ADRB3: Adrenoceptor beta 3
BAT: Black adipose tissue
BMP: Bone morphogenetic protein
cAMP: Cyclic adenosine monophosphate
CEBPB: CCAAT/enhancer-binding protein beta
CIDEA: Cell death-inducing DNA fragmentation factor-α-like effector A
CREB: cAMP response element-binding protein
FA: Free fatty acids
FGF21: Fibroblast growth factor 21
HSL: Hormone-sensitive lipase
IL-6: Interleukin-6
NE: Norepinephrine
NRG4: Neuregulin 4
OAB: Overactive bladder
PET-CT: Positron emission tomography-computed tomography
PGC-1α: PPAR-γ co-activator
PKA: Protein kinase A
PPARG: Peroxisome proliferator-activated receptor gamma
PPAR-γ: Peroxisome proliferator-activated receptor gamma
TG: Triacylglycerol
TMEM26: Transmembrane protein 26
TZD: Thiazolidinedione
UCP1: Uncoupling protein-1
VEGF: Vascular endothelial growth factor
WAT: White adipose tissue
β3-AR: Beta 3 adrenoceptor
MAPK: Mitogen-activated protein kinase
ATF-2: Activating transcription factor 2
FNDC5: Fibronectin type III domain-containing protein 5
PRDM16: Positive regulatory domain containing 16
C/EBP: CCAAT enhancer-binding protein
LBD: Ligand binding domain
Apo-CIII: Apolipoprotein CIII
HbA1c: Hemoglobin A1c
KEGG: Kyoto Encyclopedia of Genes and Genomes
